# Supplementary material for: Persistent COVID-19 Symptoms Minimally Impact the Development of SARS-CoV-2-Specific T Cell Immunity
Source: Viruses. 2021 May 15;13(5):916. doi: 10.3390/v13050916 (PMC8155927; doi:10.3390/v13050916)
Supplement: Supplementary file 1 [file viruses-13-00916-s001.zip › viruses-1214835-for conversion-suppl/Supplemental_Figures.pdf]

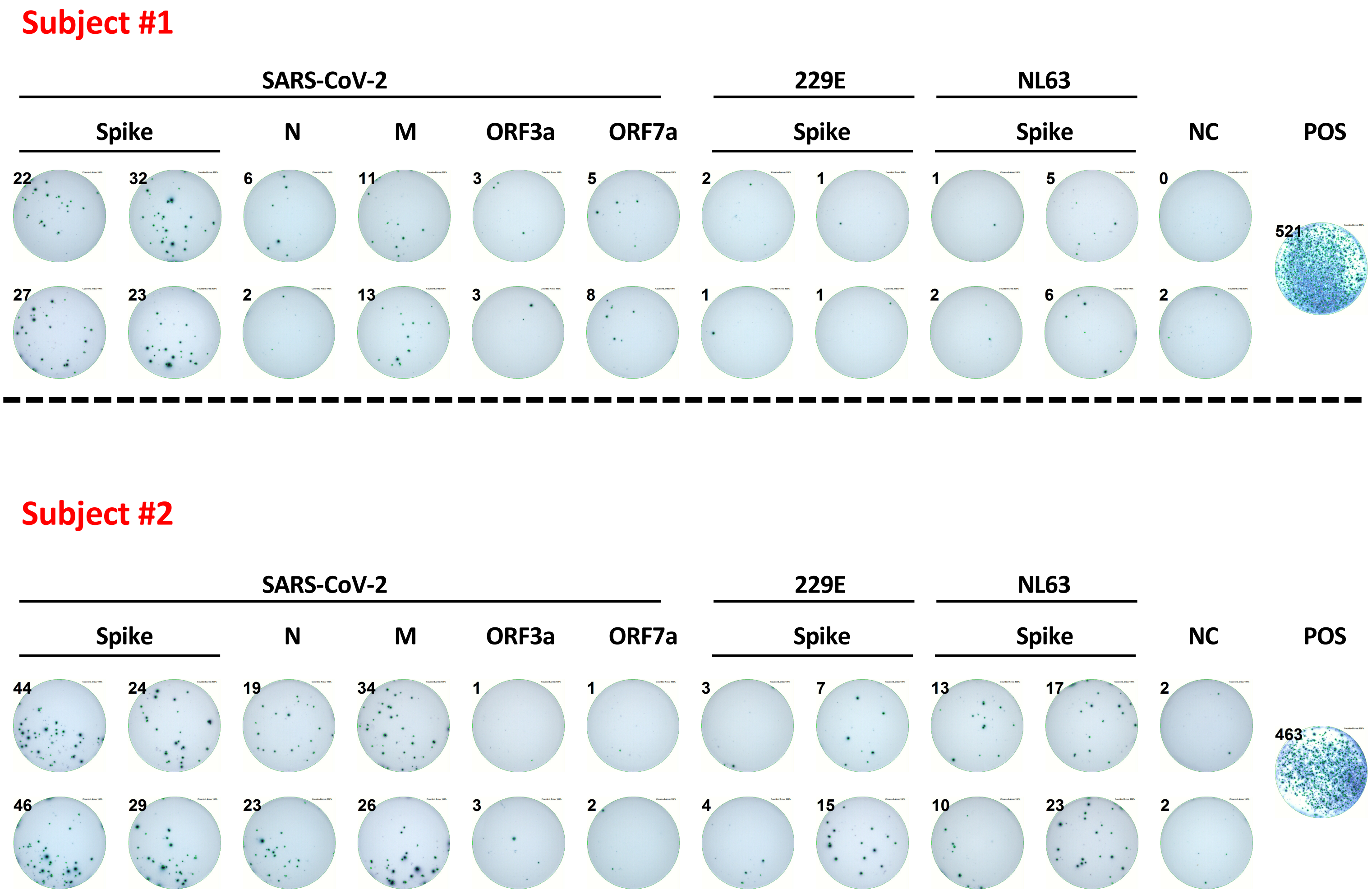

Figure S1. Representative IFN-γ ELISPOT plate images

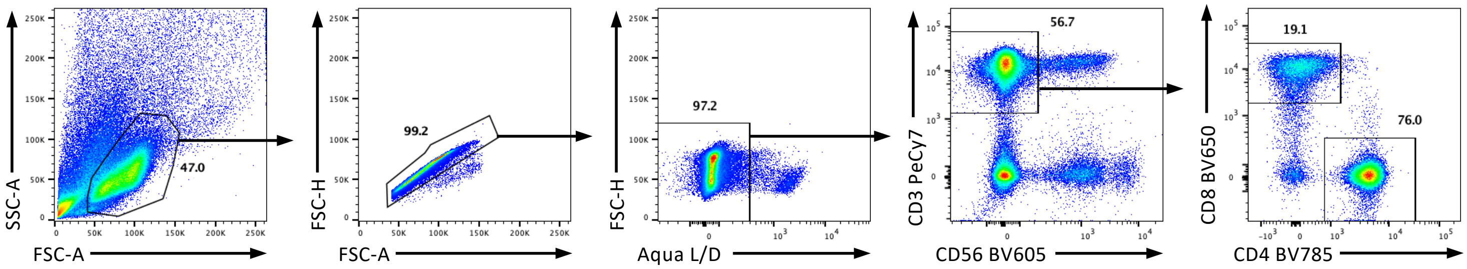

Figures S2. Gating scheme for activated CD4 and CD8 T cell isolation

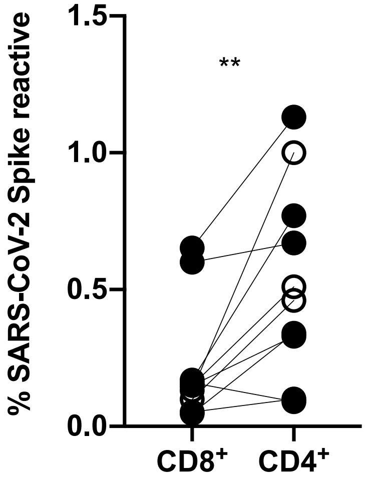

Figure S3. Background-subtracted frequency of SARS-CoV-2 Spike reactive CD8+ and CD4+ T cells in 10 select convalescent COVID-19 patients. ** $p < 0.01$ two-tailed paired T test.

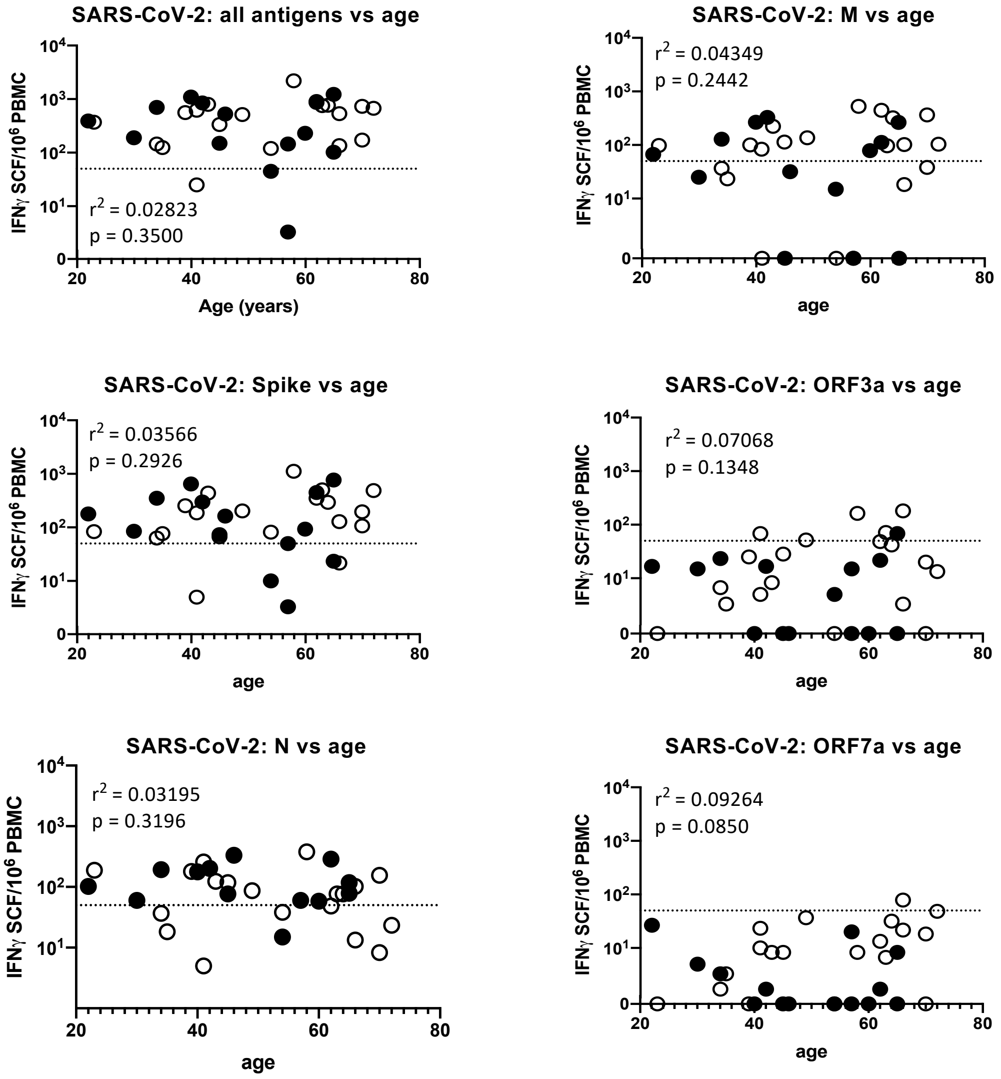

Figure S4. Relationship between age and SARS-CoV-2 antigen reactivity as assessed by IFN-g ELISPOT. Pearson correlation calculations were used to determine r2 and two-tailed p values. Dashed line indicates the 50 IFN-γ SFC/10^6 PBMC threshold for positivity
