# Supplementary material for: Persistent COVID-19 Symptoms Minimally Impact the Development of SARS-CoV-2-Specific T Cell Immunity
Source: Viruses. 2021 May 15;13(5):916. doi: 10.3390/v13050916 (PMC8155927; doi:10.3390/v13050916)
Supplement: Supplementary file 1 [file viruses-13-00916-s001.zip › viruses-1214835-for conversion-suppl/Supplemental_Tables.pdf]

Table S1. Peptide pools used for IFN-γ ELISPOT analysis

Virus	Antigen	Company	Product #
SARS-CoV-2	Spike glycoprotein	JPT Peptide Technologies	PM-WCPV-S
SARS-CoV-2	Nucleoprotein peptide pool	JPT Peptide Technologies	PM-WCPV-NCAP
SARS-CoV-2	Membrane peptide pool	JPT Peptide Technologies	PM-WCPV-VME
SARS-CoV-2	ORF3A peptide pool	JPT Peptide Technologies	PM-WCPV-AP3A
SARS-CoV-2	ORF7A peptide pool	JPT Peptide Technologies	PM-WCPV-NS7A
HCoV-229E	Spike glycoprotein	JPT Peptide Technologies	PM-229E-S-1
HCoV-NL63	Spike glycoprotein	JPT Peptide Technologies	PM-NL63-S-1

Table S2. Antibody used for flow cytometry

Antibody	Clone	Manufacturer	Catalog #	Lot #	Dilution used
CD25 PE	BC69	Biolegend	302606	B296102	1:160
CD3 PeCy7	HIT3a	Biolegend	300316	B293468	1:320
CD69 AF647	FN50	Biolegend	310918	B275384	1:160
CD134 FITC	Act36	Biolegend	350006	B296280	1:160
CD56 BV605	5.1H11	Biolegend	362537	B320741	1:160
CD8 BV650	RPA-T8	Biolegend	301041	B275821	1:320
CD4 BV785	RPA-T4	Biolegend	300553	B298451	1:160

Table S3. RNAseq metrics

Sample ID	Duration of symptoms (days)	Number of sorted cells	RNA recovery	# aligned reads
Short #1	2	3,179	2,370pg	45.6M
Short #2	5	4,536	3,255pg	47.0M
Short #3	3	648	496pg	43.6M
Short #4	5	1,318	1,170pg	48.1M
Short #5	7	4,076	2,325pg	46.0M
Long #1	19	2,214	1,095pg	45.1M
Long #2	18	1,586	765pg	45.2M
Long #3	33	4,497	2,025pg	49.6M
Long #4	61	1,061	1,155pg	46.6M
Long #5	37	106	690pg	45.0M
